# Using cocrystals as a tool to study non-crystallizing mol­ecules: crystal structure, Hirshfeld surface analysis and com­putational study of the 1:1 cocrystal of (*E*)-*N*-(3,4-di­fluoro­phen­yl)-1-(pyridin-4-yl)methanimine and acetic acid

**DOI:** 10.1107/S2053229624005187

**Published:** 2024-07-05

**Authors:** Addi Dana Sánchez-Pacheco, Eduardo H. Huerta, Josué Benjamín Espinosa-Camargo, Evelyn Valeria Rodríguez-Nájera, Diego Martínez-Otero, Simón Hernández-Ortega, Jesús Valdés-Martínez

**Affiliations:** a Instituto de Química, Universidad Nacional Autónoma de México, Circuito Exterior, Ciudad Universitaria, 04510 Coyoacán, Cd. Mx., Mexico; b CCIQS UAEM-UNAM, Universidad Nacional Autónoma de México, Carretera, Toluca-Atlacomulco Km. 14.5, Unidad San Cayetano, Toluca, 50200, Estado de México, Mexico; c Tecnológico de Estudios Superiores de Ixtapaluca, Km 7 Carretera Ixtapaluca, Coatepec, CP 56580, Ixtapaluca, Estado de México, Mexico; Universidade Federal de Minas Gerais, Brazil

**Keywords:** crystal structure, cocrystal, hydrogen bonding, π–π inter­actions, CrystalExplorer, AIM, NCI, methaninine, acetic acid

## Abstract

Using a 1:1 cocrystal of (*E*)-*N*-(3,4-di­fluoro­phen­yl)-1-(pyridin-4-yl)methanimine with acetic acid, C_12_H_8_F_2_N_2_·C_2_H_4_O_2_, we investigate the influence of F atoms introduced to the aromatic ring on promoting π–π inter­actions. The cocrystal crystallizes in the triclinic space group *P*1. Through crystallographic analysis and com­putational studies, we reveal the mol­ecular arrangement within this co­crystal, demonstrating the presence of hydrogen bonding between the acetic acid mol­ecule and the pyridyl group, along with π–π inter­actions between the aromatic rings. Our findings highlight the importance of F atoms in promoting π–π inter­actions without necessitating full halogenation of the aromatic ring.

## Abstract 2

Using a cocrystal of a mol­ecule that does not crystallize, we reveal that π–π inter­actions between aromatic rings are present with only two F atoms in the aromatic ring.

## Introduction

Understanding inter­molecular inter­actions is fundamental to designing and synthesizing functional solid-state materials. Although there are significant advances in our understanding, there is still much to com­prehend (Brammer, 2017 ▸; Galek *et al.*, 2014 ▸; Gunawardana & Aakeröy, 2018 ▸). Our research in­ves­tigates small mol­ecules derived from Schiff bases having an aromatic ring (^F^Ar) and a pyridyl group (py). These mol­ecules can form three different types of inter­molecular inter­actions: hydrogen bonds (H-bonds), inter­actions between the aromatic rings (π–π and C—H⋯π) and halogen bonds (X-bonds) when F, Br or I atoms are present. We have introduced F atoms to the Ar ring (^F^Ar) to increase the likelihood of π-inter­actions between the aromatic rings. We are looking to understand how the number and position of F atoms in ^F^Ar affect the inter­actions and organization of the mol­ecules in the crystal. Previous studies have indicated that the perfluorinated ^F^Ar ring inter­acts with the py ring through π–π inter­actions in both Schiff base (Jaime-Adán *et al.*, 2024 ▸) and alkene analogue mol­ecules [Cambridge Structural Database (CSD; Groom *et al.*, 2016 ▸) refcodes ADUJOA (Orbach *et al.*, 2012 ▸), EQOTOU (Mondal *et al.*, 2011 ▸), EQOTOU (Lucassen *et al.*, 2005 ▸) and RIDMOH (Aakeröy *et al.*, 2007 ▸)], while non-fluorinated or mono-fluorinated rings of the Schiff base and the analogue alkene only present C—H⋯π inter­actions. We aim to investigate how many F atoms are necessary to promote π–π inter­actions.

Despite our best efforts, we were unable to crystallize di­sub­stituted com­pounds successfully. However, we did manage to obtain a cocrystal of (*E*)-*N*-(3,4-di­fluoro­phen­yl)-1-(pyridin-4-yl)methanimine (DFPPI) with acetic acid (AcOH), which is an acid that does not contain aromatic rings that may inter­fere with the possible aromatic inter­actions. In this article, we present the crystal structure of the 1:1 DFPPI–AcOH co­crystal, (**1**) (Scheme 1), and reveal the inter­actions that govern its stability through Hirshfeld surface analysis and com­putational methodologies.
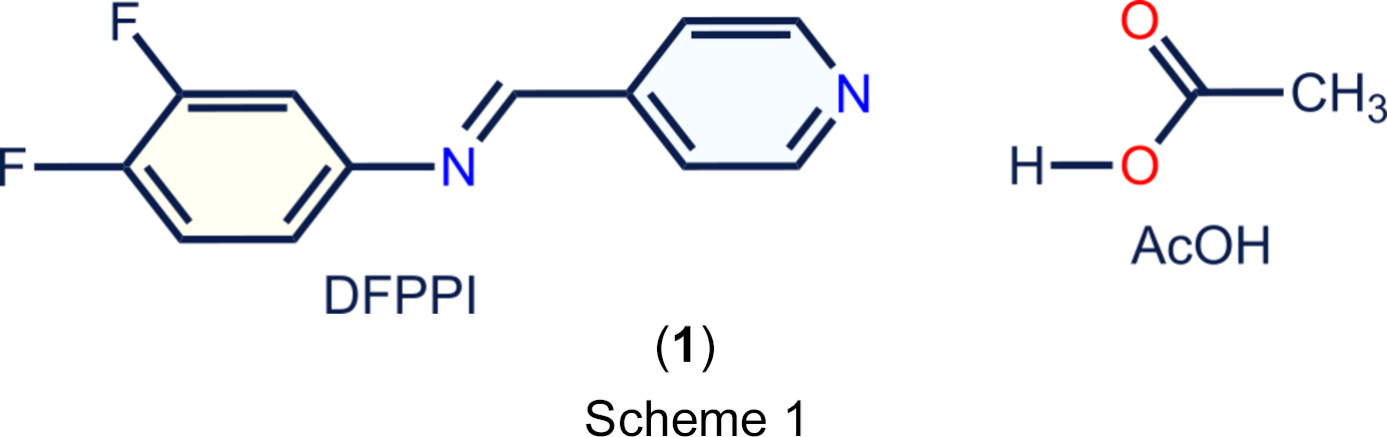


## Experimental

All solvents, starting materials and carb­oxy­lic acids were purchased from commercial sources and used without further purification. IR data were collected using a Nicolet 380 FT–IR instrument. The melting point (uncorrected) was determined using a Fischer–Johns Mel-Temp melting-point apparatus.

### Synthesis and crystallization

DFPPI was obtained from an equimolar reaction of pyridine-4-carbaldehyde and 3,4-di­fluoro­aniline as reported pre­viously (Sánchez-Pacheco *et al.*, 2021 ▸). Crystals of (**1**) were obtained from a 9:1 (*v*/*v*) ethanol–acetic acid solution as a cream–yellow powder (m.p. 340–342 K). FT–IR (ATR) ν_max_: 3058, 3030, 1627, 1597, 1107 cm^−1^. ^1^H NMR (CDCl_3_, 300 MHz): δ 8.78 (*dd*, *J* = 6.0, 2.6 Hz, 2H), 8.43 (*s*, 1H), 7.74 (*dd*, *J* = 6.0, 2.7 Hz, 2H), 7.26–7.17 (*m*, 1H), 7.16–7.08 (*m*, 1H), 7.05–6.97 (*m*, 1H). DART+, *m*/*z*: 220, 219.

### Refinement

Crystal data, data collection and structure refinement details are summarized in Table 1 ▸. Carbon-bound H atoms were placed in calculated positions and included in the refinement in the riding-model approximation, with *U*_iso_(H) values set to 1.2*U*_eq_(C). In the final analysis, we explored the isotropic displacement parameter refinement of the O and N atoms, but the results were not significant, so thermal anisotropy was applied. The oxygen-bound H atom was located from a difference Fourier map and refined with *U*_iso_(H) = 1.5*U*_eq_(O).

### Computational studies

The analysis of electron density and inter­action energies aims to discern the nature and strength of inter­actions within the cocrystal. The study began with calculating the Hirshfeld surface (Spackman *et al.*, 2021 ▸), where the *d*_norm_ and shape index (S) were then mapped to identify inter­molecular inter­actions and detect π–π inter­actions, respectively. Afterward, pairwise inter­action energies were com­puted to qu­antify inter­action strength, and an energy framework was derived to characterize the stabilizing inter­actions within the network. Both the calculation of the Hirshfeld surface and the energy analysis were conducted in *CrystalExplorer*, employing the CE-B3LYP/6-31G(d,p) level with *TONTO* (Jayatilaka & Grimwood, 2003 ▸; Turner *et al.*, 2015 ▸; Mackenzie *et al.*, 2017 ▸). Further insights into the inter­actions were achieved through theoretical electron-density analysis using the GPUAM code (Cruz *et al.*, 2019 ▸; Hernández-Esparza *et al.*, 2014 ▸, 2018 ▸), which combines two methodologies, namely, the Quantum Theory of Atoms in Mol­ecules (QTAIM) and the Non-Covalent Inter­actions (NCI) Index. The theoretical electron density was generated using *GAUSSIAN16* [B3LYP/6-31G(d,p)] (Frisch *et al.* 2016 ▸).

## Results and discussion

**Figure 1:**
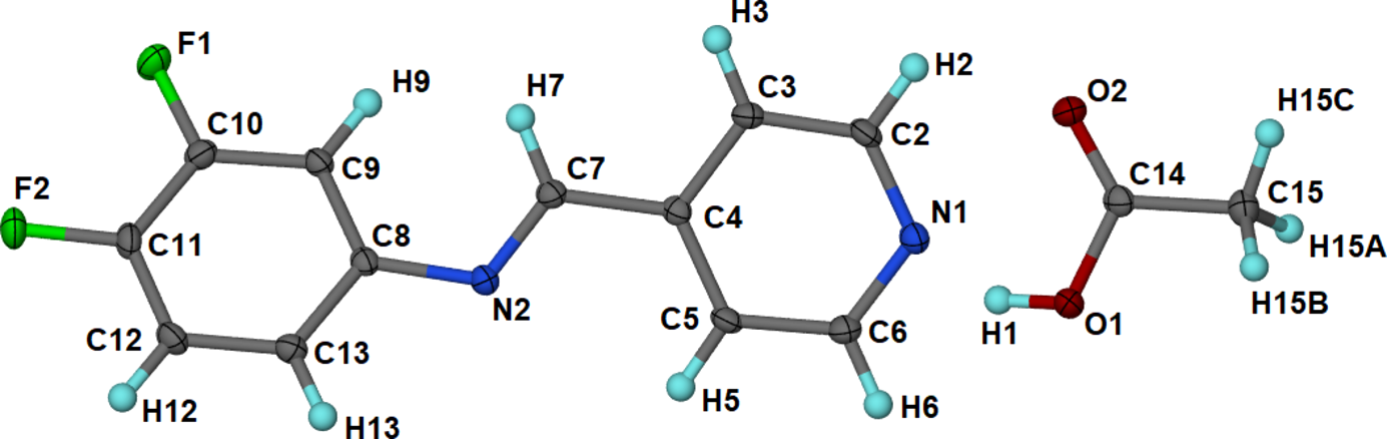
The asymmetric unit of cocrystal (**1**), showing the atom-numbering scheme. Displacement ellipsoids are drawn at the 50% probability level and H atoms at an arbitrary size.

Cocrystal (**1**) consists of one DFPPI mol­ecule and one acetic acid mol­ecule in its asymmetric unit (Fig. 1 ▸). The crystal system is triclinic and belongs to the space group *P*1. The imine group has an *E* conformation. The DFPPI mol­ecule is not planar, as evidenced by the relevant torsion angles (Table 2 ▸) and the dihedral angle of 29.89 (5)° between the planes of the pyridine (py) and aromatic (^F^Ar) rings. The AcOH mol­ecule shows C—O distances according to single and double C—O bonds, in agreement with the presence of an acid group and not a carboxyl­ate, as expected for a cocrystal. The C—O bonds in the AcOH mol­ecule are nearly in the same plane as the py ring. This is confirmed by the angle formed between the py ring and the heavy atoms of AcOH, which measures 10.80 (6)°.

**Figure 2:**
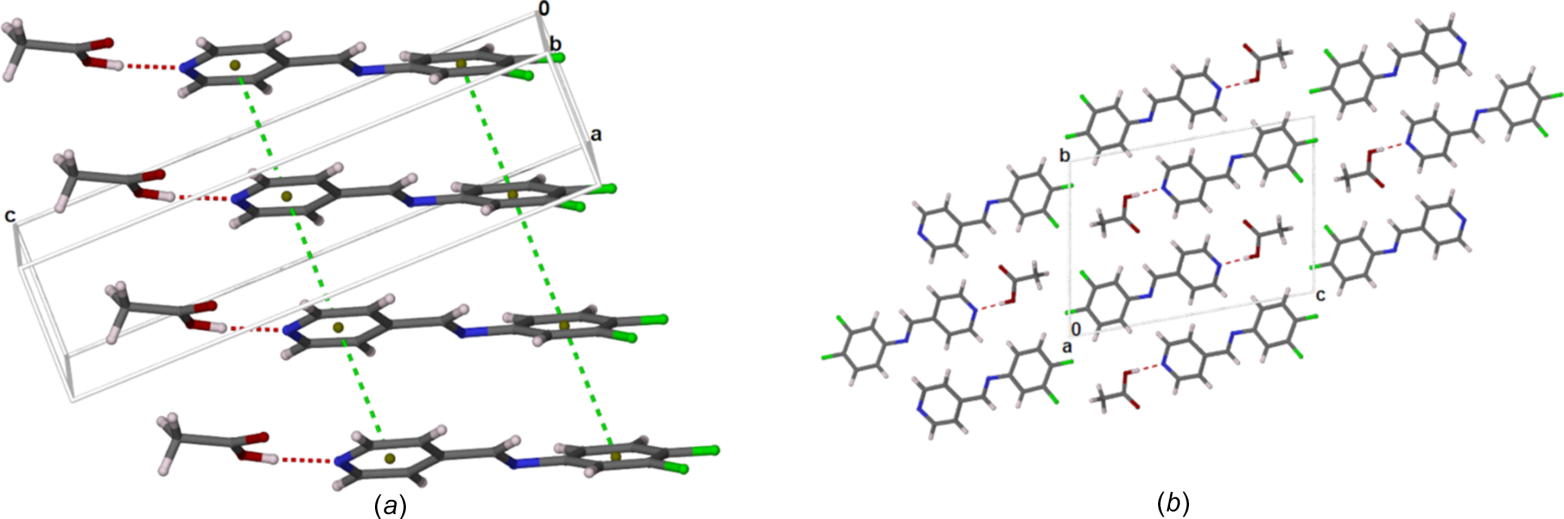
The inter­molecular inter­actions in (**1**). Hydrogen bonds are indicated as red dashed lines and π–π inter­actions as green dashed lines. (*a*) View of the mol­ecules organized in columns through π–π inter­actions. (*b*) The packing of mol­ecules along the *a* axis, showing the O—H⋯N(py) hydrogen bonding.

The AcOH mol­ecule forms an O1—H1⋯N1^i^ hydrogen bond with the N1 atom of the py from the imine (Table 3 ▸). Moreover, it shows py⋯py and ^F^Ar⋯^F^Ar π–π inter­actions, with centroid–centroid distances of *Cg*(py)⋯*Cg*(py) = 3.8047 (6) Å and *Cg*(^F^Ar)⋯*Cg*(^F^Ar) = 3.8047 (7) Å; these inter­actions organize the mol­ecules in columns [Fig. 2 ▸(*a*)] and the columns close pack to build the crystal [Fig. 2 ▸(*b*)].

**Figure 3:**
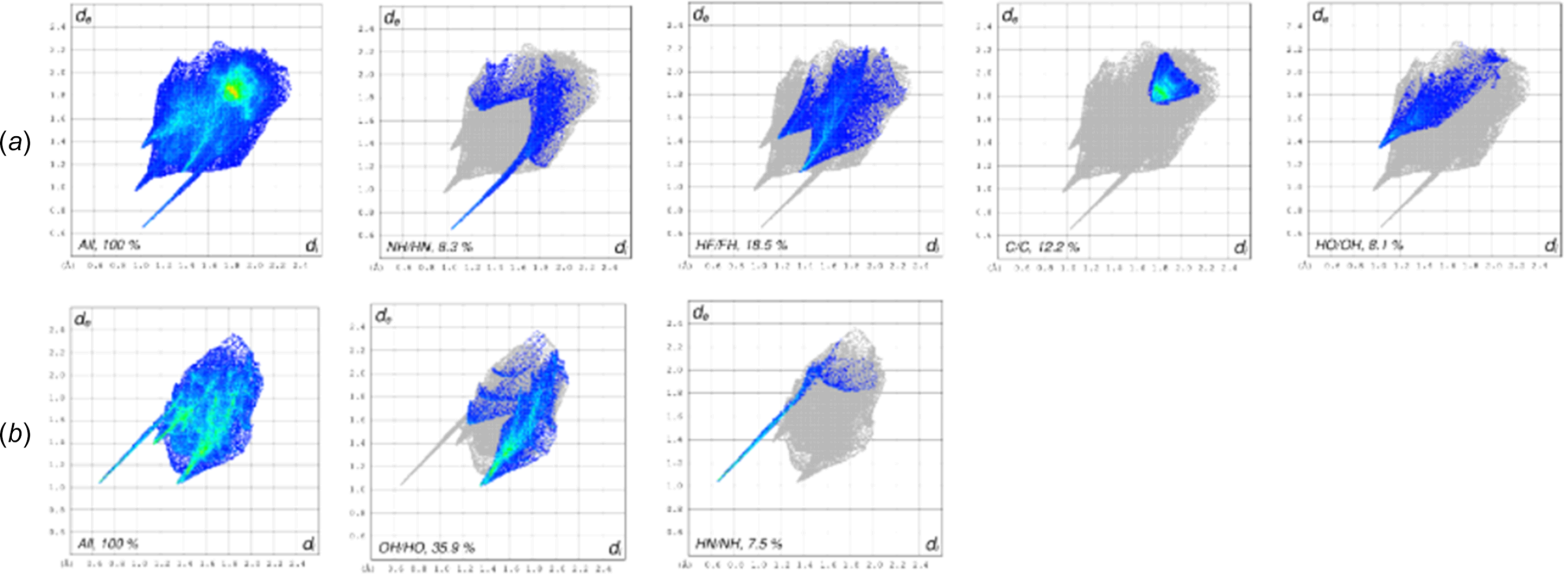
Selected 2D fingerprint plots for (*a*) (*E*)-*N*-(3,4-di­fluoro­phen­yl)-1-(pyridin-4-yl)methanimine (*b*) and acetic acid in (**1**).

We used the *CrystalExplorer* program to generate Hirshfield surfaces and mapped them with *d*_norm_ and shape index, and two-dimensional (2D) fingerprints to determine the inter­molecular inter­actions (McKinnon *et al.*, 2007 ▸). Fig. 3 ▸ shows the 2D fingerprints of DFPPI and AcOH. The plots show the typical wing structures with a non-symmetric long pick, which corresponds to the N1⋯H1 interaction on the DFPPI mol­ecule and H1⋯N1 in the AcOH mol­ecule, corresponding to the O1—H1⋯N1^i^ hydrogen bond between both mol­ecules. There is another hydrogen bond, namely, C2—H2⋯O2^ii^, *i.e.* C2—H2⋯O2 in DFPPI and O2⋯H2—C2 in AcOH. Additionally, the fingerprint of DFPPI indicates bonds of the type C—H⋯F and inter­actions between the C atoms, suggesting π–π inter­actions.

**Figure 4:**
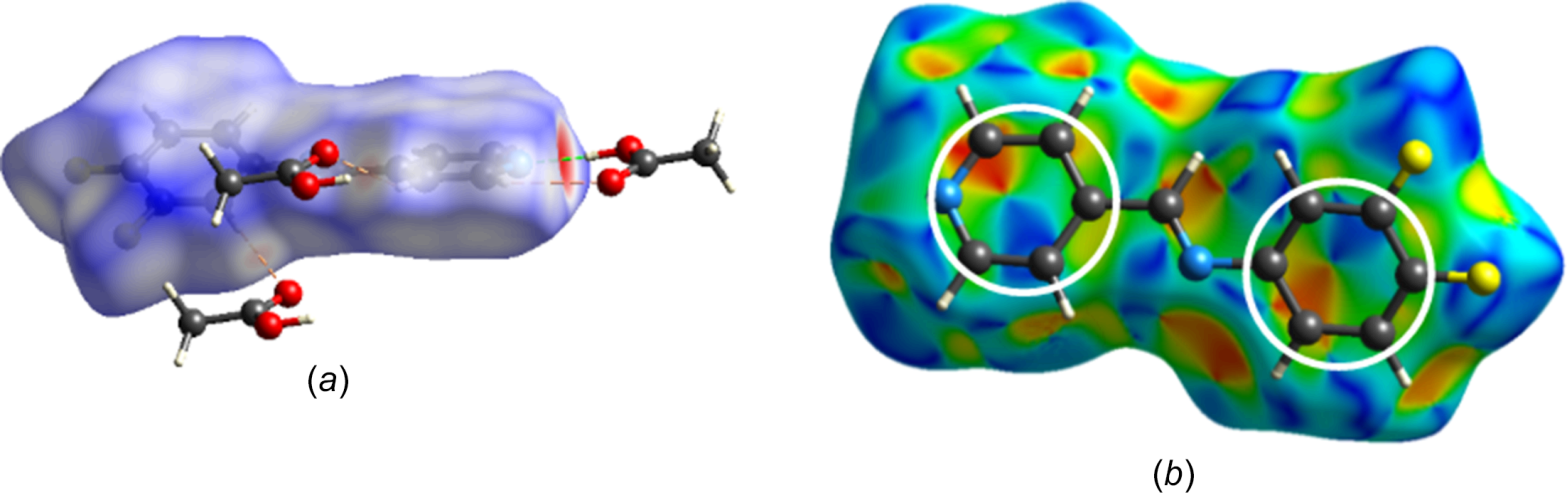
Hirshfeld surface mapped with (*a*) *d*_norm_, with the hydrogen bonds between mol­ecules, and (*b*) shape index. The red and blue triangles inside the rings agree with the presence of π–π inter­actions.

Fig. 4 ▸(*a*) displays the Hirshfeld surface, mapped with *d*_norm_, which shows the existence of O—H⋯N(py) and C—H⋯O hydrogen bonds. Fig. 4 ▸(*b*) shows the Hirshfeld surface mapped with shape index; the com­plementary blue and red triangles observed in the aromatic rings indicate the presence of π–π inter­actions between the ^F^Ar and py rings (McKinnon *et al.*, 2004 ▸).

**Figure 5:**
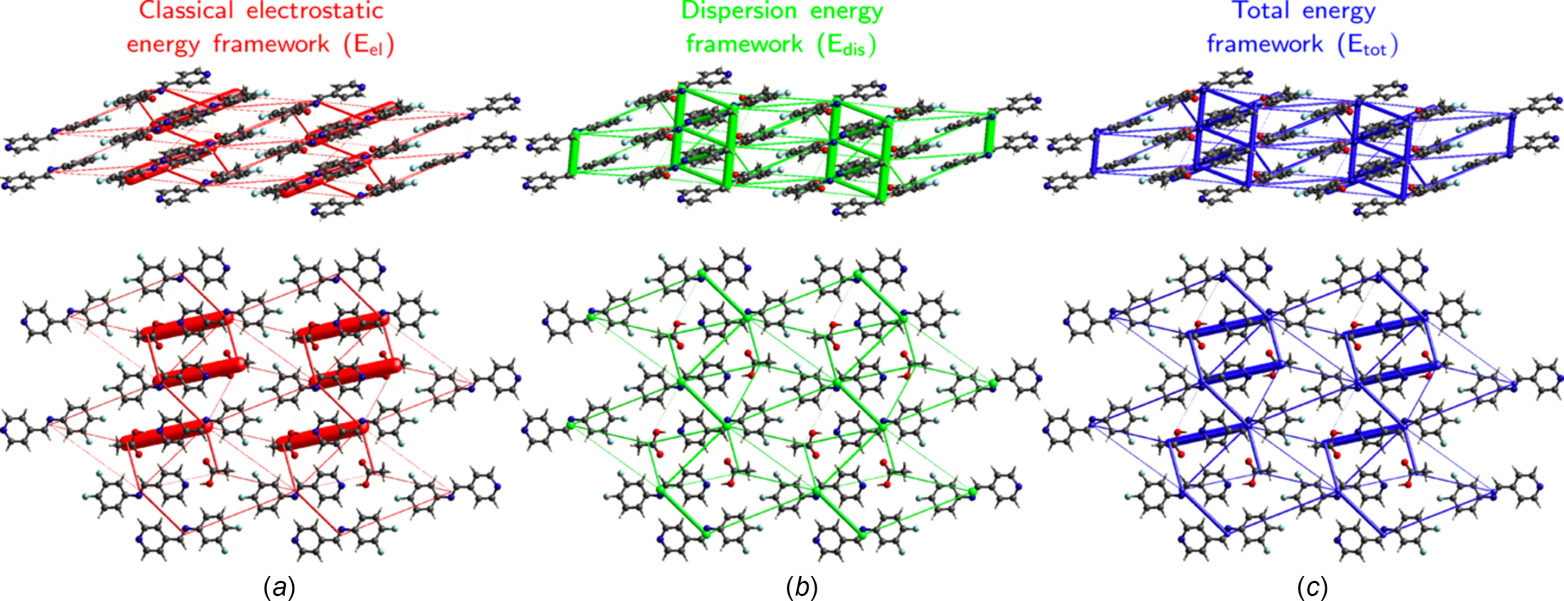
Perspective and top views of the energy frameworks of the cocrystal, showing the (*a*) electrostatic energy, (*b*) dispersion energy and (*c*) total energy. The radius of the cylinders is proportional to the relative strength of the corresponding energies. They were adjusted to the same scale factor of 80 with a cut-off value of 0 kJ mol^−1^ within a 2 × 2 × 2 unit cell.

Table 4 ▸ presents selected results from the calculation of pair­wise inter­action energies relative to the DFPPI mol­ecule, along with a colour-coded mol­ecular cluster illustrating these inter­actions. As expected, the most robust inter­action, highlighted in red, was observed between the DFPPI mol­ecule and the AcOH mol­ecule. This inter­action involves a hydrogen bond between O—H(acid) and N(py), with a total inter­action energy (*E*_tot_) of −49.4 kJ mol^−1^. The inter­actions between DFPPI molecules stacked on top of each other, coloured in green in Table 4 ▸, follow in energy. According to the Hirshfeld surface, this inter­action represents π–π inter­actions between the aromatic rings; the aryl and pyridine rings inter­act with an energy of −31.1 kJ mol^−1^. The cocrystal network seems sig­nificantly influenced by other inter­actions, including those between DFPPI mol­ecules that do not have π–π characteristics. Non-classical hydrogen-bond contacts like C(imine)—H⋯O(acid) and C(ar­yl)—H⋯O(carbon­yl) also play a role in the inter­actions between the DFPPI and AcOH mol­ecules. Finally, the rod-shaped energy frameworks (Fig. 5 ▸) highlight that the stability of the cocrystal is governed by multiple electrostatic forces, with dispersive inter­actions having an im­portant contribution, which is more significant between stacked mol­ecules.

**Figure 6:**
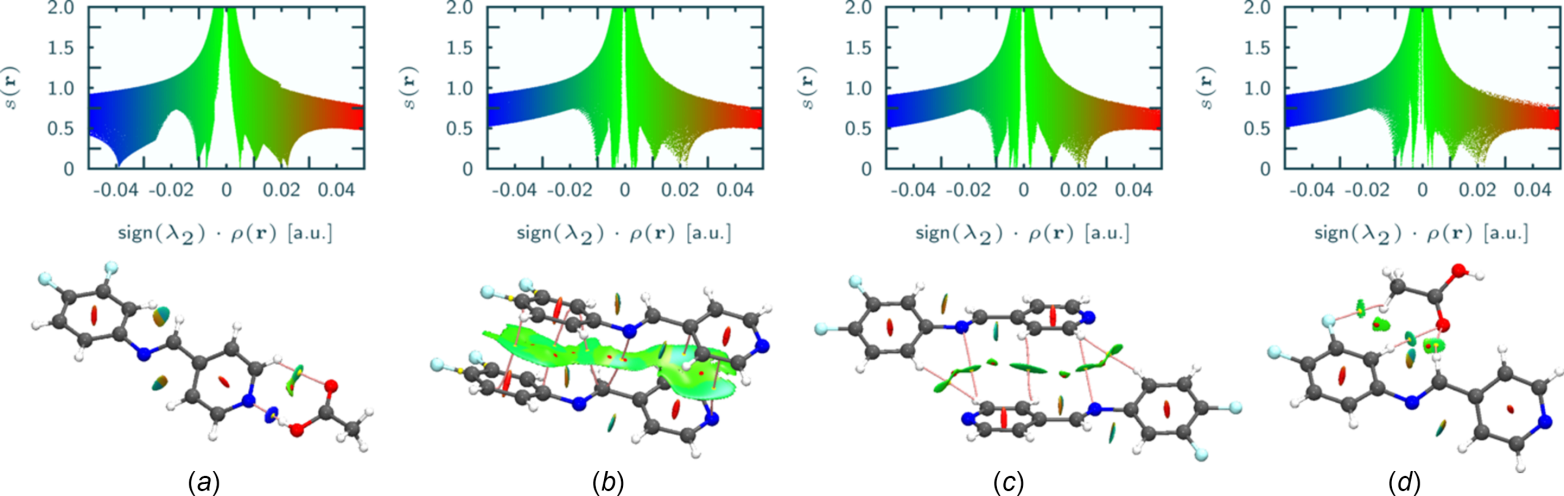
Plots of the reduced gradient of the density *s*(r) *versus* the electron density multiplied by the second Hessian eigenvalue (top) and mol­ecular diagrams with the isosurfaces (isovalue = 0.5) of the *s*(r), the bond trajectories (pink) and the critical points (yellow) that exhibit the contacts for the dimers where the inter­actions were the strongest, based on the magnitude of *E*_tot_. Parts (*a*)–(*d*) are for dimers corresponding to entries 1–4 of Table 4 ▸.

Theoretical electron-density analysis generates a spatial visualization and classifies pairwise inter­actions as attractive or repulsive (Fig. 6 ▸). Focusing on the four pairs with the most negative *E*_tot_ values, we observe bond trajectories for O—H⋯N and C—H⋯O hydrogen-bond contacts. Based on the NCI index, these inter­actions are attractive. Electrostatic inter­actions play a significant role in the total inter­action energy of mol­ecular pairs. Regarding stacking inter­actions, bonding trajectories connecting C atoms of inter­acting DFPPI mol­ecules are identifiable, accom­panied by a prominent iso­sur­face indicative of weakly attractive stacking. Such characteristics align with the heightened dispersive character sug­gested by the *E*_tot_ com­ponents for these pairs.

Our assumption that a cocrystal would help study the inter­molecular inter­actions of mol­ecules that do not crystallize was successful. We found that having two F atoms in the aromatic ring is sufficient to promote π–π inter­actions between the aromatic rings, and further halogenation of the ^F^Ar ring is unnecessary.

## Supplementary Material

Crystal structure: contains datablock(s) I, global. DOI: 10.1107/S2053229624005187/dg3053sup1.cif

Structure factors: contains datablock(s) I. DOI: 10.1107/S2053229624005187/dg3053Isup2.hkl

Supporting information file. DOI: 10.1107/S2053229624005187/dg3053Isup3.cml

CCDC reference: 2359735

## Figures and Tables

**Table 1:** Experimental details

Crystal data	
Chemical formula	C_12_H_8_F_2_N_2_·C_2_H_4_O_2_
*M*_r_	278.26
Crystal system, space group	Triclinic, *P* 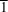
Temperature (K)	100
*a*, *b*, *c* (Å)	3.8047 (1), 11.0101 (4), 15.4968 (6)
α, β, γ (°)	79.535 (1), 89.223 (1), 82.880 (1)
*V* (Å^3^)	633.42 (4)
*Z*	2
Radiation type	Mo *K*α
μ (mm^−1^)	0.12
Crystal size (mm)	0.35 × 0.28 × 0.21

Data collection	
Diffractometer	Bruker APEXII CCD
No. of measured, independent and observed [*I* > 2σ(*I*)] reflections	11642, 2896, 2531
*R*_int_	0.083
(sin θ/λ)_max_ (Å^−1^)	0.650

Refinement	
*R*[*F*^2^ > 2σ(*F*^2^)], *wR*(*F*^2^), *S*	0.038, 0.110, 1.06
No. of reflections	2896
No. of parameters	185
No. of restraints	1
H-atom treatment	H atoms treated by a mixture of independent and constrained refinement
Δρ_max_, Δρ_min_ (e Å^−3^)	0.39, −0.28

Computer programs: *APEX2* (Bruker, 2005 ▸), *SAINT* (Bruker, 1998 ▸), *SHELXT* (Sheldrick, 2015*a* ▸), *SHELXL* (Sheldrick, 2015*b* ▸), *X-SEED 4* (Barbour, 2020 ▸), *CIFTAB* (Sheldrick, 2008 ▸) and *PLATON* (Spek, 2020 ▸).

**Table 2:** Selected geometric parameters (Å, °)

O1—C14	1.3243 (14)	N2—C7	1.2739 (15)
O2—C14	1.2166 (14)	N2—C8	1.4188 (13)
			
C7—N2—C8	119.44 (9)	O2—C14—C15	123.74 (10)
N2—C7—C4	120.72 (10)	O1—C14—C15	112.80 (9)
O2—C14—O1	123.43 (10)		
			
C3—C4—C7—N2	−179.29 (10)	C7—N2—C8—C13	−151.63 (11)
C5—C4—C7—N2	0.01 (17)	C7—N2—C8—C9	30.40 (16)

**Table 3:** Hydrogen-bond geometry (Å, °)

*D*—H⋯*A*	*D*—H	H⋯*A*	*D*⋯*A*	*D*—H⋯*A*
O1—H1⋯N1^i^	0.86 (1)	1.83 (1)	2.6819 (12)	174 (2)
C2—H2⋯O2^ii^	0.95	2.64	3.3344 (14)	130
C3—H3⋯O2^iii^	0.95	2.48	3.3174 (14)	147
C9—H9⋯O2^iv^	0.95	2.56	3.5088 (14)	173
C13—H13⋯O1^v^	0.95	2.65	3.3713 (14)	134
C15—H15*B*⋯F2^vi^	0.98	2.61	3.5224 (14)	155

Symmetry codes: (i) 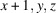; (ii) 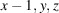; (iii) 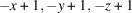; (iv) 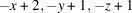; (v) 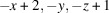; (vi) 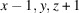.

**Table 4:** Pairwise inter­action energy analysis using B3LYP/6-311G(d,p) as the energy model The energies (*E*) are in kJ mol^−1^ and the radial distance (*R*) in Å. The colour-coded mol­ecular cluster is related to the specific inter­action energy.
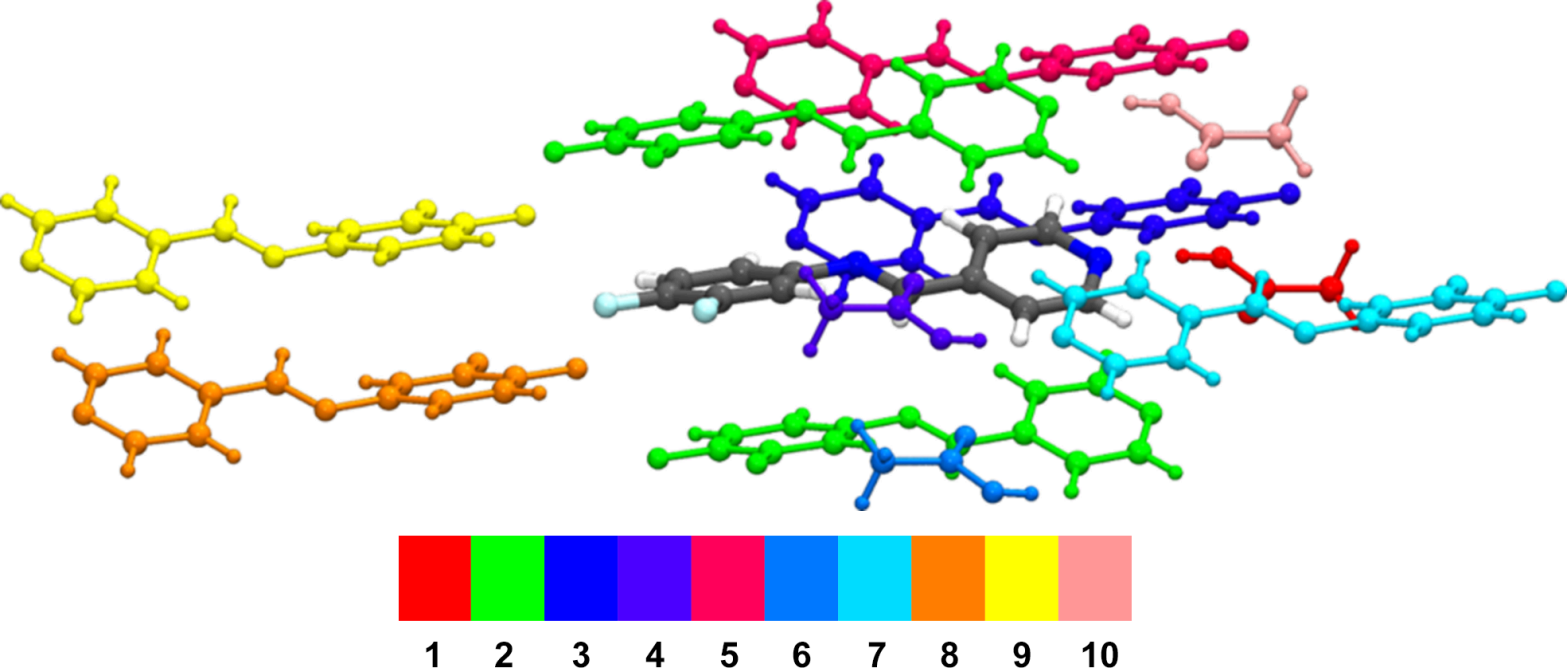

	No.	Symop	*R*	*E*_ele_	*E*_pol_	*E*_dis_	*E*_rep_	*E*_tot_	*E*	*E*_BSSE_
**1**	1	–	8.79	−81.5	−18.8	−11.5	98.2	−49.4	−53.9	−41.8
**2**	2	*x*, *y*, *z*	3.80	0.3	−1.1	−59.0	32.0	−32.1	−51.5	−33.6
**3**	1	−*x*, −*y*, −*z*	7.90	−9.9	−1.1	−24.5	18.1	−21.4	−34.6	−24.3
**4**	1	–	4.76	−12.3	−3.3	−12.2	11.2	−19.2	30.7	−20.7
**5**	1	−*x*, −*y*, −*z*	7.22	−9.1	−1.3	−24.1	22.4	−17.7	−31.2	−23.6
**6**	1	–	5.08	−9.1	−1.3	−24.1	22.4	−17.7	−31.2	−23.6
**7**	1	−*x*, −*y*, −*z*	10.07	−4.4	−0.9	−14.2	13.0	−9.7	−15.6	−11.4
**8**	1	−*x*, −*y*, −*z*	10.81	−4.0	−0.5	−8.8	5.2	−9.0	−19.1	10.6
**9**	1	−*x*, −*y*, −*z*	11.57	−3.4	−0.4	−7.3	3.0	−8.4	−17.11	−9.54
**10**	1	–	8.14	−2.0	−0.6	−5.1	0.9	−6.4	−9.9	−6.7
Scale factors for benchmarked energy model										
Energy model				*k*_ele_	*k*_pol_	*k*_dis_	*k*_rep_			
CE-B3LYP-B3LYP-D2/6-31G(d,p)				1.057	0.740	0.871	0.618			
